# Winter’s Toll: Assessing the Public Health Impact of Cold-Related Illnesses and Energy Damage in Ukraine During the Invasion

**DOI:** 10.21203/rs.3.rs-6589736/v1

**Published:** 2025-06-04

**Authors:** Kimia Marvi, Iftikhar U Sikder, Shanshan Wang, Juan Espinoza, Nancy Fiedler, Julia Pavlova, Irina Holovanova, Emily S. Barrett, Ubydul Haque

**Affiliations:** Independent Consultant; Cleveland State University; University of North Texas Health Science Center; Stanley Manne Children’s Research Institute, Ann & Robert H. Lurie Children’s Hospital of Chicago; Rutgers University; Lviv State University of Physical Culture; Poltava State Medical University; Rutgers University; Rutgers University

**Keywords:** Russian invasion, Public health, Energy infrastructure strike

## Abstract

During the Russian invasion of Ukraine, targeted attacks on energy infrastructure exposed civilians to heightened cold-related health risks. This study aimed to: (1) characterize respiratory infections and cold-related injuries during the conflict; (2) identify vulnerable sociodemographic groups; (3) assess household adaptations; and (4) evaluate how winter preparation influenced health outcomes. We surveyed 2,311 households across 24 Ukrainian oblasts during the 2022–2023 winter. One adult per household provided data on demographics, winter preparations, housing, heating, and access to services. Machine learning models were used to predict respiratory infections, symptoms, and cold injuries, based on sociodemographic and household factors. Respiratory infections affected 75.2% of participants, and 3.76% reported cold injuries—rising to 10% among older adults. Larger households experienced more respiratory infections, while Russian-controlled areas reported higher cold injury rates. Key predictors of respiratory infections included age, household size, financial stability, and heating practices; cold injuries were predicted by age, region, anxiety, and household size. This is the first study to apply machine learning to examine cold-related health impacts following energy infrastructure attacks in an active conflict zone. Our findings underscore the vulnerability of older adults and the widespread burden of respiratory infections, highlighting the need for targeted cold injury prevention in conflict-affected and cold-climate regions.

## Background

The Russian invasion that began in February of 2022 not only resulted in deaths and injuries but also impacted the lives and safety of Ukrainian civilians who experienced “wide area effects,” like shelling, multiple-launch rocket systems, artillery, missiles, and air strikes.^1,2^ The coverage highlights the challenges of dealing with the multifaceted nature of warfare, which affects both rural and urban areas through various battles. As the conflict progressed, Russian strategy evolved to include launching missiles, rockets, and loitering munitions, targeting energy facilities.^3^ These facilities provide heating and electricity indispensable for survival, including water supplies, domestic heating, hospitals, and other critical infrastructure facilities such as street lighting, communication systems, and banking systems. Russian attacks are estimated to have severely damaged 40% of Ukraine’s thermal generation, 90% of its wind power, and over 40% of its solar and nuclear energy sources, which were either under occupation or damaged.^3^ These relentless assaults on the power grid have resulted in millions of Ukrainians experiencing shortages of electricity, heat, water, and other essential services.^3^ While such disruptions pose an inconvenience in summer months, which typically have a humid continental climate with warm summers, they are most dangerous in winter, when temperatures are typically −3 to −6 °C, but can drop as low as - sometimes they drop to −20° or even −30 °C.^4^

Conflict and forced displacement have long been linked to respiratory infections among displaced populations.^5^ However, the deliberate destruction of civilian energy infrastructure during harsh winters presents an unprecedented threat, significantly heightening the risk of cold-related injuries and respiratory illnesses. To our knowledge, no prior studies have examined the impact of such targeted attacks on civilian health during a war, highlighting a critical gap in understanding the full extent of suffering inflicted in these conditions. Here, cold injury refers to tissue damage caused by exposure to cold.^6^ However, it is not the only factor determining injury severity. Cold injury occurs when the body’s ability to adapt to low temperatures is compromised, influenced by exposure duration, humidity, wind, altitude, clothing, medical conditions, and individual variability. Cold injuries can be pre-freezing (trench foot and chilblains/pernio) or freezing (hypothermia and frostbite) and can affect specific body parts or the entire body.^6^ The respiratory infections and symptoms of a viral infection affecting the nose, sinuses, throat, and windpipe are distinct from influenza. Flu is also an infection of the nose, throat, and lungs, part of the respiratory system.

Given the nature of an unprecedented attack on energy infrastructure during the winter season, it is imperative to understand the public health impact of cold injuries and the adaptation and mitigation of the affected population to reduce injuries and infections. Thus, there is a clear need to conduct a study on respiratory infections and symptoms and cold injuries.

Specifically, we sought to: (1) characterize respiratory infections and cold-related injuries among Ukrainians during the Russian invasion. We hypothesized that households with children and older adults in crowded conditions would face a heightened risk of respiratory infections and cold injuries due to limited heating and access to medical care. (2) Identify sociodemographic groups most at risk of cold-related illness, with the hypothesis that psychological distress and financial instability would increase vulnerability by limiting individuals’ ability to adopt preventive behaviors and access essential resources. (3) Understand how Ukrainians have adapted to changing conditions (e.g., energy savings, homelessness), hypothesizing that those engaging in proactive energy-saving behaviors (e.g., layering clothing, using alternative heating) would report fewer cold-related health outcomes. (4) Evaluate whether winter preparation at the individual and household levels mitigated adverse health effects, hypothesizing that effective preparation would be especially protective for vulnerable populations facing infrastructure failure and extreme cold.

## Methodology

### Data Collection and Preparation:

TGM Research, a nationally representative survey panel in Ukraine, recruited participants from April 5, 2023, to May 15, 2023, via emails, in-app notifications, or text messages. Using a quota sampling design, a representative sample was obtained from Ukraine’s 26 administrative regions, including 24 oblasts and two special status cities (Kyiv City and Sevastopol City).^7^ The online survey data were collected from one adult per household aged 18–72 in 2311 households across 24 oblasts in Ukraine.

All methods were performed in accordance with the relevant guidelines and regulations.

Demographic data were collected on participants’ age, sex, internally displaced population (IDP) status, education, marital status, and occupation (Tables S1-S16). The questionnaire gathered data on respondents’ living and health conditions, identifying diseases like respiratory infections and symptoms, cold injuries, heart attacks, etc. (Tables S1-S16) during the winter season from November 2022 to April 2023. The dataset included health issues among all household members, including internally displaced persons living in the same household, children (<18 years), older adults (>60 years), and pregnant individuals within the household, along with the overall health status of the household. A final list of features are provided in the supplement (Excel data file 1).

Participants responded to 39 yes/no questions about whether they had experienced any of the following cold-related conditions during the Russian invasion. When they endorsed yes, they were asked, ‘How many people in your home experienced the event?’ and ‘Do they have access to the medications or treatments of the diseases or symptoms?’. Self-reported respiratory infections and symptoms, including flu, pneumonia, rhinorrhea, cough, sneeze, fever, and sore throat, were merged due to the small positive numbers. Participants reported hypothermia, trench or immersion foot, chilblain, frostbite, frostnip, Raynaud’s disease, and cornea freezing. Due to the relatively small number of participants reporting hypothermia, trench or immersion foot, chilblain, frostbite, frostnip, Raynaud’s disease, and cornea freezing we created a dichotomous cold injuries variable, indicating whether anyone in the household, including vulnerable members (such as children, pregnant women, and older adults), experienced any cold injuries during October 2022 to March 2023.

### Preprocessing:

The collected data underwent preprocessing to ensure its quality and suitability. We received 2,311 survey responses. 183 (7.9%) surveys represent missing values in the survey (e.g., 999 household size). Because we set a forced choice, participants could not skip questions. Participants were asked to report ‘999’ if they did not know the answer. For the ML models, these values were all replaced with −1. For statistical analysis, these surveys were censored. Another 5 (0.2%) responses had household sizes ranging from 50 to 300, likely representing individuals in shelters.

### Statistical analysis:

We performed a Chi-Square and T-test on the characteristics of survey respondents. We first applied traditional statistical methods. However, these approaches yielded weak results due to the dataset’s complexity, which included 140 features across various sub-groups, such as pregnant women, children, older adults, and other household members. Traditional methods required extensive feature engineering and struggled to capture relationships among multiple variables, leading to information loss. In contrast, machine learning effectively analyzed the full dataset, automatically identifying key features, validated models through high-performance metrics, and provided deeper insights into health outcomes.^8^

These values were all replaced with 55 for the ML models and used in the ML analyses. Due to scarcity, there were 17 (0.7%) responses, which we did not include in the Chi-Square and T-test. In the end, 2,111 (91.3%) responses were included in the statistical analysis and visualizations in Figures S1-S4.

The dataset was categorized into 140 independent variables (features) and target variables (outcome variables) for effective modeling. Feature engineering was used to identify and target variables related to cold injuries. To simplify classification, we analyzed two outcomes -respiratory infections and symptoms, and cold injuries - by converting the variables into binary values.

### Feature selection:

The experiments involved implementing models based on features such as demographic, socio-economic, living conditions, access to basic amenities, and behavioral factors, as listed in Tables S1-S16. The feature importance ranges from 0 to 1, with higher values indicating greater significance. Our model retained features that exceeded a threshold of 0.02. The best models were evaluated for feature importance, with variables such as those that were not biologically or environmentally plausible removed to enhance performance. The iterative feature selection process aimed to improve the models’ performance and interpretability by eliminating irrelevant features without negatively impacting performance.

### ML models:

ML models were used to predict respiratory infections and cold injuries and, more importantly, identify the most relevant features and their impact on these outcomes. The choice of ML over traditional statistical models was based on the complexity of the data, which involved numerous features. ML models are better suited for handling large datasets and capturing intricate relationships between variables, which can be difficult for traditional methods to model effectively (7).

In this study, we applied the ML techniques Decision Tree (DT), Random Forest (RF), XGBoost, AdaBoost, Support Vector Machine (SVM), and Multi-layer Perceptron (MLP) for analysis.^9,10^ After ensuring the models were reliable, feature importance was calculated using the mean decrease in impurity (MDI), which helps identify the key variables contributing to the outcomes (21). The evaluation metrics and hyperparameter tuning methods used to assess model reliability are described below.

Due to the imbalanced dataset, with a high prevalence of common cold and a low prevalence of cold injuries, models may be biased towards the majority class, achieving high accuracy by predicting it consistently. To assess model performance comprehensively, we report multiple evaluation metrics. While accuracy is often used as a standard metric, it can be misleading in imbalanced datasets, as high accuracy can be achieved by predicting the majority class more frequently without properly identifying the minority class. To address this limitation, we also report precision, recall, F1-score, and confusion matrix, which provide a more balanced view of model performance, especially for the minority class. The F1-score, in particular, is critical for evaluating models in such settings, considering false positives and false negatives. Accuracy measures the proportion of correctly classified instances out of the total instances; precision indicates the proportion of true positive results among all positive results; recall (or sensitivity) measures the proportion of true positive results out of all actual positives; and the F1-score is the harmonic mean of precision and recall. These metrics are reported in percentages, with higher values indicating better performance. The confusion matrix provides a detailed breakdown of true positive, true negative, false positive, and false negative classifications.

The study utilized hyperparameter optimization to identify the most effective hyperparameter combination for each ML model, a crucial process for enhancing model performance. One standard method for hyperparameter optimization is grid search^9^, which systematically explores a predefined grid of hyperparameters and evaluates the model performance for each combination. Grid search is an algorithm that specifies hyperparameters and their possible values, trains the model using each combination, and evaluates its performance through cross-validation. The model’s optimal set of hyperparameters results in the highest performance, which we measured by F1-score.

The study used a 5-fold cross-validation strategy to assess the models during the grid search. The approach divides the dataset into five folds, using four for training and the remaining for validation, repeating the process five times. The model’s overall performance is evaluated by calculating the average performance across all folds. This study employed hyperparameter optimization and cross-validation to ensure the robustness of the selected models, enabling their ability to generalize to unseen data effectively.

## Results

Respiratory infections and symptoms were prevalent (75.2%), especially in larger households and among adults aged 30–44 (78%) or 60+ (80%) (Table S11/Figure S1–2). Cold injuries affected 3.76% of participants overall, but 10% of older adults (Table S7–8). Households with children or older adults had higher infection rates (Figure S5-S7). Six Oblasts had an increase in the number of households since the start of the invasion (11.4–18.6%), while others declined in household number (10–100%) (Table S3). Nearly 50% of participants lost heating equipment during the invasion (Table S8), and 29% reported lacking necessary medicine (Table S6). Most respondents from Russian-occupied areas moved after the invasion and tended to have higher cold injury rates compared to non-occupied areas (Figure S4).

**Figure 1:**
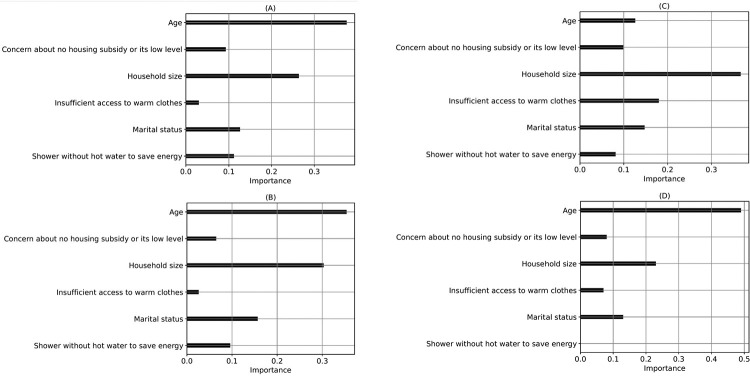
The importance of each input feature in respiratory infections and symptoms prediction at the household level (all population) using various ML algorithms. (A) DT, (B) RF, (C) XGBoost, (D) Adaboost (feature name on the left side).

To evaluate the significance of independent features, other than the involvement of older adults and children in the household, in predicting respiratory infections and symptoms at the household level, considering all population groups, we experimented with predicting this target without using those two features as inputs. Table 1 presents the classification performance of different ML algorithms for this experiment. The evaluation metrics include accuracy, precision, recall, and F1-score, with results averaged over a 5-fold cross-validation. AdaBoost outperformed all models, while XGBoost had the highest F1 score (Table 1). The study emphasizes the significance of feature selection and the influence of specific features on predictive performance. Figure 1 highlights the role of each input feature in predictive models, revealing the connections between these features and the occurrence of respiratory infections and symptoms in households. Results consistently indicated that age and household size were the most significant features in predicting seasonal respiratory infections and symptoms, as per DT, RF, XGBoost, and Adaboost models. However, the XGBoost model prioritized “household size” as the most crucial factor in predicting seasonal respiratory infections and symptoms, surpassing “age”. ML algorithms differed in key predictors, revealing varied patterns in household respiratory infection and symptom risks (Figure 1). The number of true positives (correctly predicted occurrences of seasonal respiratory infections and symptoms) was high across all models, while false positives and true negatives were very close (Figure S8).

**Figure 2:**
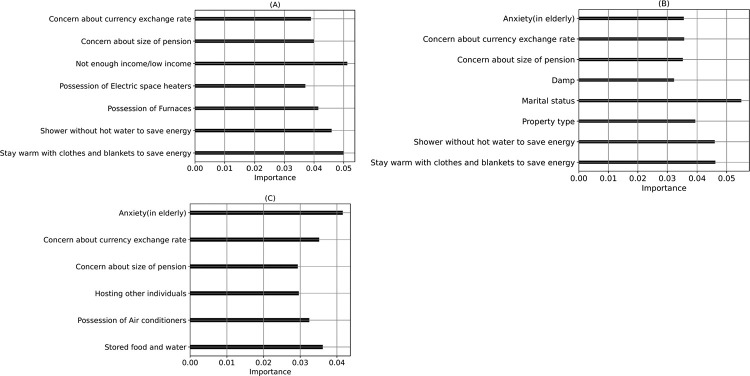
The most important and plausible features in cold injury prediction in older adults (>60 years) using various ML algorithms. (A) DT, (B) RF, (C) XGBoost, (D) Adaboost (feature name on the left side).

The algorithms, including RF and XGBoost, showed high accuracy and F1-score in predicting seasonal respiratory infections and symptoms among older adults (Table 2). Financial stability significantly influenced the likelihood of experiencing seasonal respiratory infections and symptoms, with concerns about the currency exchange rate, pension size, and low-income levels emerging as impactful features. In addition, “Anxiety (in older adults)” was identified as another influential feature shared between RF and XGBoost, highlighting its role in predicting seasonal respiratory infections and symptoms. DT and RF models highlighted the significance of energy-saving behaviors like showering without hot water and staying warm with clothes and blankets in predicting seasonal respiratory infections and symptoms among older adults (Figure 2). The models show a high sensitivity to seasonal respiratory infections and symptoms, with many true positives and fewer true negatives (Figure S9). Out of the 1045 households including older adults, only 207 had not experienced respiratory infections and symptoms (19.8%, Figure S9).

**Figure 3:**
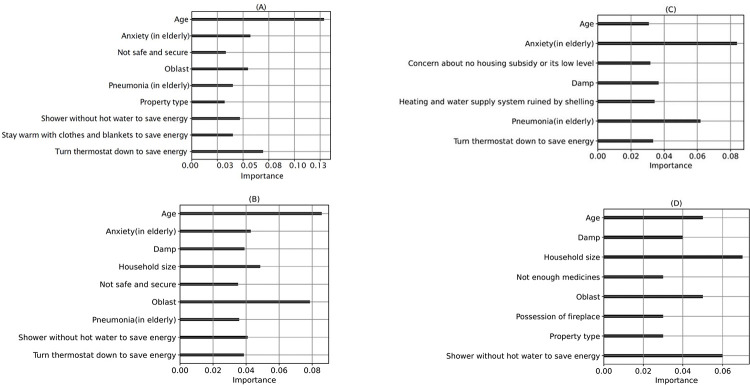
The most important and plausible features in respiratory infections and symptoms prediction in older adults (>60 years) using various ML algorithms. (A) DT, (B) RF, (C) XGBoost.

XGBoost demonstrated superior accuracy and precision in predicting cold injuries among older adults, while AdaBoost achieved the highest F1 score (Table 3). Across DT, RF, and Adaboost models, “Age” emerged as the most crucial feature for predicting cold injuries in older adults, indicating its significant impact on the outcome. Additionally, the models highlighted the importance of “Oblast” (region) and “Household size” in predicting cold injuries. In contrast, XGBoost ranked “Anxiety (in older adults)” as the most influential feature, followed by “Pneumonia (in older adults)” and “Damp,” indicating its unique ranking compared to other models (Figure 3). The study suggests high specificity in predicting cold injuries, consistent with high true negatives and low true positives in all models (Figure S10). Only 10% of older adults experienced cold injuries. The presence of children or the elderly increased infections, driving 90.4% model accuracy (Table S18).

### Model validation

To ensure the robustness and reliability of our ML models, we employed rigorous model validation techniques, including hyperparameter optimization and 5-fold cross-validation. Hyperparameter optimization, particularly through grid search, allowed us to fine-tune the models by identifying the most effective parameter combinations, enhancing their performance. The 5-fold cross-validation provided a comprehensive assessment of model performance by partitioning the data into five subsets, ensuring that each model was trained and validated on different data splits. This process mitigated the risk of overfitting and provided a more accurate estimate of how the models would perform on unseen data. A small sample of positive samples and a high number of input features in predictions for older adults increase the risk of overfitting. In contrast, predicting respiratory infections at the household level, with a larger positive sample size and fewer input features, reduces this risk. The consistency of high accuracy, precision, recall, and F1 scores across different models and datasets, as demonstrated in Tables 1, 2, and 3, underscores the reliability of our findings. The low standard deviation in performance metrics across the folds further confirms the stability and generalization capability of the models. These validation methods confirm that our models accurately predicted seasonal respiratory infections, symptoms, and cold injuries with high sensitivity and specificity across various household conditions.

## Discussion

In this study of Ukrainians surveyed during the Russian invasion, over 80% of respondents reported respiratory infections and symptoms, and 5% reported cold injuries in their households. The prevalence of cold injuries was 10% among the older adult population (>60 years). Nearly 50% of respondents reported that their heating equipment had been destroyed during the crisis, and 13.7% faced difficulties obtaining wood or fuel for their homes due to severe winter conditions caused by the extensive damage to energy infrastructure from Russian attacks.

In war-frontline oblasts such as Cherkasy and Ivano-Frankivsk, respiratory infections and symptoms were positively associated with household size, likely due to mass refugee movements and poorer living conditions with limited access to medical care and necessities. Among participants in this sample, six Oblasts (Cherkasy, Ivano-Frankivsk, Poltava, Ternopil, Zakarpattia, Zhytomyr) saw an increase in households ranging from 11.4% to 18.6% during the invasion, while another six Oblasts (Donetsk, Kharkiv, Kherson, Kyiv, Luhansk, and Zaporizhia) decreased in total households ranging from 10% to 100%.

Considering the pre-Russian invasion^11,12^, our findings suggested that 75.2% of participants were infected by respiratory infections and symptoms, meaning infections were most common among people aged 30–44 (78%) who worked outside of the home and also had a child or older adults in the household. Household size was a key feature of seasonal respiratory infections and symptoms; overcrowded homes may result from hosting other families, potentially increasing the opportunity for transmission of infection among residents. Different ML algorithms predicted seasonal respiratory infections and symptoms occurrences in households differently, with all models generally predicting positive associations between these household characteristics and infections, especially for older adults.

Our findings suggest concerns about currency exchange rates, pension size, and low income among older adults may have hindered effective cold prevention measures, which may have increased their risk of respiratory infections and symptoms. As suggested by the World Bank’s report^13^ and other studies.^14^ Many direct and indirect associations can be made here: having a high pension means having access to money, insurance, and other resources to alleviate illnesses. It is also indicative of having greater stability.^13^ Factors like food storage, furnace, and electric heater possession can reduce anxiety while showering without hot water.^15^ Not wearing warm clothes and staying without blankets can increase respiratory infections and symptoms.

Our results showed a high incidence of cold injuries among older individuals from Donetsk, Kherson, Luhansk, and Zaporizhia during the Russian invasion. This may be attributable to failure to leave their homes despite the compromised energy grid, as reported elsewhere.^16,17^ It may also reflect relocation to avoid attacks during the cold, snowy winter season; prior literature demonstrates that older adults are particularly at risk of cold injuries due to decreased body temperature regulation and reduced heat energy production.^18^

Our findings highlight the critical role of housing conditions in health outcomes during conflicts.^1^ Patients with cold injuries were more likely to live in poorly heated homes, particularly in oblasts near conflict zones. Published literature suggests warmer indoor environments could reduce cold injuries and lower respiratory infection risks.^19^ Another study suggests cold and damp conditions promote viral and bacterial spread, increasing pneumonia risks.^20^ Additionally, anxiety and poor living conditions may further exacerbate cold injuries in older adults.^21,22^ These results emphasize the need for improved housing and heating access to enhance health resilience in conflict-affected regions.

Consistent with Hryhorczuk et al.^23^ findings during the invasion in Ukraine, our findings highlight the impact of damaged heating infrastructure on cold injuries. The destruction of Ukraine’s fireplaces, heating, and water systems likely contributed to increased cold injuries. Our study reinforces the strong link between heating access and cold injury risk, suggesting that a functioning fireplace may be a crucial protective factor against freezing temperatures in affected households.^23^

Using ML approaches allowed us to significantly improve, particularly AdaBoost and XGBoost, which significantly improve risk prediction for cold injuries and respiratory infections, enabling more precise prevention strategies for vulnerable populations. XGBoost’s ability to handle structured medical data establishes it as a powerful tool for epidemiological modeling, while its feature importance rankings highlight key risk factors like temperature exposure and comorbidities. These insights optimize resource allocation and intervention efforts.

The study also leveraged iterative learning and built-in regularization methods to strike an optimal balance between bias and variance, which is crucial in real-world scenarios with imperfect data. The analysis confirms the superior performance of boosting techniques in the specific context by systematically evaluating multiple algorithms. It provides valuable benchmarks for selecting and fine-tuning models for similar problems. This comparative assessment underscores the significance of algorithmic selection and parameter optimization, offering insights to inform future research and practical implementations in environments characterized by challenging data conditions.

The models generally predicted respiratory infections and symptoms with high true positive rates but also showed increased false positives due to inherent uncertainties. The high-performance metrics in older adults were significantly influenced by the high prevalence of seasonal respiratory infections and symptoms, which affected over 80% of individuals. The models showed high specificity in predicting cold injuries, with high true negatives and low true positives, suggesting a higher likelihood of negative outcomes in older adults. The study highlights the difficulty in predicting cold injuries in older adults, as evidenced by poor performance metrics and low recall scores across all models.

To the authors’ knowledge, this study is the first to investigate respiratory infections and symptoms and cold injuries among non-military personnel in a war setting, using comprehensive questionnaires to gather detailed information. The study highlights the use of ML models for disease forecasting, enhancing interpretability, and facilitating the processing of large quantities of features.^24,25^ Recognizing cold-related injuries, illnesses, and symptoms is crucial for public health actions. Identifying at-risk population subgroups and factors can help prevent or manage most adverse health impacts.^26^

Although we strove to obtain a representative sample of age and sex, we had an underrepresentation, and there was a risk of sampling bias due to fewer rural participants, potentially leading to lower incidents of local cold injuries. Reliance upon self-reported health data may introduce bias, potentially affecting the generalizability of the results. Medical diagnoses of cold injury were self-reported and could not be confirmed through medical records, which may have led to error, and indeed, the model had low sensitivity. Our analysis focused on the observed health outcomes during the war. Still, without a baseline comparison, it is impossible to establish a clear causal link between the war and the infections. Other unmeasured factors could also have contributed to the observed cold-related health concerns.

While the models performed reasonably well in terms of overall accuracy and F1-score for the prediction of respiratory infections in both the household level (Table 1) and the older adults (Table 2), the significant class imbalance in the dataset likely contributed to the low F1-score for the prediction of cold injuries in the older adults (Table 3). The model’s tendency to predict the majority class more often resulted in poor performance for the minority class.

Although 5-fold cross-validation was used to evaluate model performance, the relatively small sample size in predictions done on the older adults, combined with a relatively high number of input features, increased the risk of overfitting. In contrast, the prediction of respiratory infections at the household level (all population) with a larger sample size and fewer input features presented a lower risk of overfitting.

Future studies with more balanced and representative data will be important to validate further and refine these findings, especially to ensure their applicability to broader, more diverse populations.

## Conclusion

Here, we identified risk factors that elevated the chances of respiratory infections, symptoms, and cold injuries within households among Ukrainians during the Russian invasion. Limited efforts have been made to raise awareness about risk factors, adaptation, and resiliency among war-impacted populations, particularly families and social groups, and few studies have considered the public health impacts of energy infrastructure destruction during harsh winters and military invasions. This study highlights that all age groups are at risk, with household size, currency exchange concerns, pension size, and marital status being key features predicting respiratory infections and symptoms. For cold injuries, important features were households with residents over 60 and the presence of heating equipment. These findings can lead to effective interventions and the development of adaptation and mitigation strategies, such as cold injuries and respiratory infections, during an energy crisis in harsh winter. Deliberate attacks on energy infrastructures during the winter season may impose further trauma, increase disability, and result in mass suffering during the current Russian invasion of Ukraine, as well as during other wars set in cold or seasonally variable climates.

## Figures and Tables

**Table 1:** Classification performance of various ML algorithms on seasonal respiratory infections and symptoms at the household level (all population)

Method	Accuracy		Precision		Recall		F1-score	
	Mean	Standard Error	Mean	Standard Error	Mean	Standard Error	Mean	Standard Error
Decision Tree	69.453	0.012	76.892	0.010	75.651	0.010	76.261	0.009
Random Forest	73.519	0.005	76.456	0.004	85.523	0.007	80.725	0.004
XGBoost	74.167	0.006	76.497	0.006	86.922	0.010	81.353	0.005
AdaBoost	72.523	0.007	74.944	0.005	86.590	0.007	80.342	0.005
SVM	73.346	0.005	75.576	0.004	87.058	0.008	80.900	0.004
Neural Network	70.185	0.006	76.439	0.010	78.524	0.025	77.274	0.009

***Accuracy*:** Accuracy measures a classification model’s performance by calculating the ratio of correctly predicted instances to total cases. It provides anoverall assessment of the model’s ability to classify data correctly.

***Precision*:** Precision is a metric that evaluates how well a classification model identifies positive instances. It represents the ratio of correctly predictedpositive cases to the total number of cases predicted as positive. Precision is essential when minimizing false positives is critical.

***Recall*:** Recall measures how effectively a model captures all the relevant positive cases and is critical when the goal is to minimize the number of missedpositive instances.

***F1-score*:** The F1-score is a machine learning metric that assesses a classification model’s performance, especially in cases with imbalanced datasets, bycombining precision and recall into a single measure.

The mean values represent the average performance of the model across the 5-fold cross-validation, reported as percentages. The standard error (SE)indicates the variability of the performance measures across the different folds, with lower SE values indicating more consistent model performance. Formore details, refer to the Methods section.

**Table 2:** Classification performance of various ML algorithms on respiratory infections and symptoms among the elderly (>60 years old) population.

Method	Accuracy		Precision		Recall		F1-score	
	Mean	Standard Error	Mean	Standard Error	Mean	Standard Error	Mean	Standard Error
Decision Tree	71.196	0.015	82.624	0.004	81.142	0.022	81.803	0.012
Random Forest	80.478	0.004	81.827	0.001	97.254	0.005	88.873	0.003
XGBoost	81.053	0.002	82.789	0.002	96.421	0.003	89.085	0.001

***Accuracy*:** Accuracy measures a classification model’s performance by calculating the ratio of correctly predicted instances to total cases. It provides an overall assessment of the model’s ability to classify data correctly.

***Precision*:** Precision is a metric that evaluates how well a classification model identifies positive instances. It represents the ratio of correctly predicted positive cases to the total number of cases predicted as positive. Precision is essential when minimizing false positives is critical.

***Recall*:** Recall measures how effectively a model captures all the relevant positive cases and is critical when the goal is to minimize the number of missed positive instances.

***F1-score*:** The F1-score is a machine learning metric that assesses a classification model’s performance, especially in cases with imbalanced datasets, by combining precision and recall into a single measure.

The mean values represent the average performance of the model across the 5-fold cross-validation, reported as percentages. The standard error (SE) indicates the variability of the performance measures across the different folds, with lower SE values indicating more consistent model performance. For more details, refer to the Methods section.

**Table 3.** Classification performance of various ML algorithms on cold injuries among the elderly (>60 years old) population.

Method	Accuracy		Precision		Recall		F1-score	
	Mean	Standard Error	Mean	Standard Error	Mean	Standard Error	Mean	Standard Error
Decision Tree	69.282	0.013	30.177	0.028	26.551	0.030	27.960	0.026
Random Forest	76.555	0.010	44.802	0.123	9.681	0.024	15.683	0.037
XGBoost	77.895	0.005	52.886	0.036	18.564	0.022	27.351	0.028
AdaBoost	76.746	0.005	47.656	0.023	26.596	0.025	33.876	0.025
Neural Network	69.665	0.018	29.444	0.037	20.683	0.022	23.617	0.022

***Accuracy*:** Accuracy measures a classification model’s performance by calculating the ratio of correctly predicted instances to total cases. It provides an overall assessment of the model’s ability to classify data correctly.

***Precision*:** Precision is a metric that evaluates how well a classification model identifies positive instances. It represents the ratio of correctly predicted positive cases to the total number of cases predicted as positive. Precision is essential when minimizing false positives is critical.

***Recall*:** Recall measures how effectively a model captures all the relevant positive cases and is critical when the goal is to minimize the number of missed positive instances.

***F1-score*:** The F1-score is a machine learning metric that assesses a classification model’s performance, especially in cases with imbalanced datasets, by combining precision and recall into a single measure.

## Data Availability

Supporting data for this manuscript are included in the article and supplementary files. Original datasets are available from the corresponding author upon request.
